# Hydrogel Encapsulation of Genome-Engineered Stem Cells for Long-Term Self-Regulating Anti-Cytokine Therapy

**DOI:** 10.3390/gels9020169

**Published:** 2023-02-20

**Authors:** Kelsey H. Collins, Lara Pferdehirt, Leila S. Saleh, Alireza Savadipour, Luke E. Springer, Kristin L. Lenz, Dominic M. Thompson, Sara J. Oswald, Christine T. N. Pham, Farshid Guilak

**Affiliations:** 1 Department of Orthopaedic Surgery, Washington University, St. Louis, MO 63110, USA; 2 Shriners Hospitals for Children, St. Louis, MO 63110, USA; 3 Center of Regenerative Medicine, Washington University, St. Louis, MO 63110, USA; 4 Department of Biomedical Engineering, Washington University, St. Louis, MO 63110, USA; 5 Department of Mechanical Engineering and Materials Science, Washington University, St. Louis, MO 63110, USA; 6 Division of Rheumatology, Department of Medicine, Washington University, St. Louis, MO 63110, USA

**Keywords:** induced pluripotent stem cells, implant, drug delivery, rheumatoid arthritis, autoimmune, designer cells

## Abstract

Biologic therapies have revolutionized treatment options for rheumatoid arthritis (RA) but their continuous administration at high doses may lead to adverse events. Thus, the development of improved drug delivery systems that can sense and respond commensurately to disease flares represents an unmet medical need. Toward this end, we generated induced pluripotent stem cells (iPSCs) that express interleukin-1 receptor antagonist (IL-1Ra, an inhibitor of IL-1) in a feedback-controlled manner driven by the macrophage chemoattractant protein-1 (Ccl2) promoter. Cells were seeded in agarose hydrogel constructs made from 3D printed molds that can be injected subcutaneously via a blunt needle, thus simplifying implantation of the constructs, and the translational potential. We demonstrated that the subcutaneously injected agarose hydrogels containing genome-edited Ccl2-IL1Ra iPSCs showed significant therapeutic efficacy in the K/BxN model of inflammatory arthritis, with nearly complete abolishment of disease severity in the front paws. These implants also exhibited improved implant longevity as compared to the previous studies using 3D woven scaffolds, which require surgical implantation. This minimally invasive cell-based drug delivery strategy may be adapted for the treatment of other autoimmune or chronic diseases, potentially accelerating translation to the clinic.

## 1. Introduction

Biologic therapies have revolutionized treatment options for rheumatoid arthritis (RA). However, RA and autoimmune diseases typically manifest as dynamic flares of pain and inflammation, while the clinical standard for treatment involves continuous administration of biologic drugs at high doses [1,2,3,4]. In this respect, continuously high levels of cytokine inhibition have been associated with a number of adverse events, including increased risk of infection and potential cancer development [5]. These drawbacks have prompted the development of improved drug delivery systems that can sense and respond proportionally to disease flares [1,4].

As such, we previously developed implantable anti-cytokine therapeutics that can sense and respond to dynamic changes in endogenous levels of inflammation to overcome the challenges of current biologic treatment [6]. One approach to addressing these issues has been to use cells as implantable drug delivery systems with prescribed stimulus-response systems reprogrammed into the genome. In previous studies, we combined the principles of synthetic biology with CRISPR-Cas9 genome engineering to create self-regulating induced pluripotent stem cells (iPSCs) that rapidly produce cytokine-inhibiting biologic drugs when stimulated by transient inflammatory signals [1,4]. Specifically, these “smart” cells respond to interleukin-1 (IL-1) by transcribing interleukin-1 receptor antagonist (IL-1Ra, an inhibitor of IL-1) or luciferase control (Luc) in a feedback-controlled manner driven by the macrophage chemoattractant protein-1 (Ccl2) promoter [1,4]. We demonstrated that surgically implanted 3D woven scaffolds [7,8] seeded with genome engineered Ccl2-IL1Ra cells mitigated disease activity and completely prevented bone erosions in the K/BxN serum-induced arthritis model [6]. However, these scaffolds contained relatively low cell densities and required an invasive surgical procedure for subcutaneous implantation, potentially limiting the translational potential of this approach. 

In the present study, reprogrammed self-regulating iPSCs were embedded at high concentration in agarose, a marine-derived polysaccharide hydrogel with thermogelling behavior, which has been established as a biocompatible hydrogel delivery system [9,10]. Cell encapsulation in soft hydrogels such as agarose is a key strategy for overcoming some of the existing limitations of cell-based drug delivery kinetics [11,12,13], including rapid clearance and low cell viability, especially in the context of cancer immunotherapies [14] and biologics for rheumatic or autoimmune diseases [15]. Agarose readily supports chondrogenesis [1,4,10,16,17,18,19,20,21,22,23,24] and has been used as an injectable hydrogel, but can also be shaped a priori to provide precise control over implant geometry [14]. Here, we tested the ability of cell-laden agarose rod-shaped cartilaginous implants, produced using 3D printed molds and injected subcutaneously with a wide bore needle to provide a long-term stable drug depot, similar to the longevity provided by the rod-shaped birth control implants [25]. We hypothesized that the delivery of high cell densities via an injectable implant would provide a desirable alternative to invasive surgical implantation of such hydrogel or 3D woven scaffold systems [6]. Such an approach could provide the ease of implantation while maintaining or enhancing therapeutic efficacy. Furthermore, the development of this translational delivery strategy promises to overcome the limitations of the surgical implantation approach and can be applied to a variety of disease processes beyond RA. 

## 2. Results

### 2.1. Initial Agarose Construct Testing 

**Figure 1:**
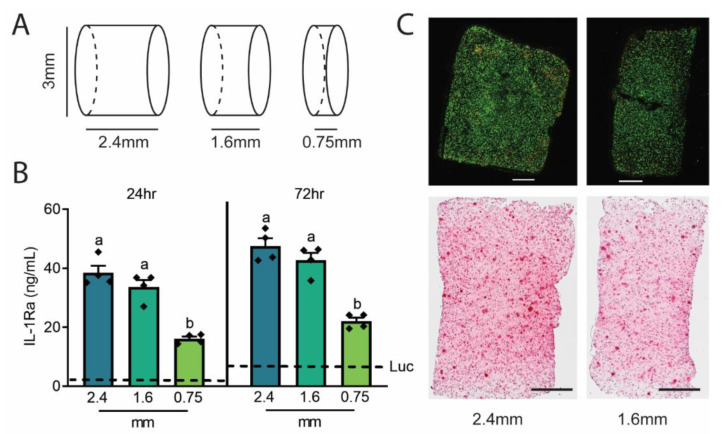
(**A**) Schematic of agarose constructs of different thicknesses (2.4 mm [surface area = 36.8 mm^2^], 1.6 mm [29.2 mm^2^], and 0.75 mm [21.2 mm^2^]) loaded with Ccl2-IL1Ra iPSCs at the same cell densities and stimulated for chondrogenesis for 3 weeks. (**B**) After 24 h and 72 h of 1 ng/mL IL-1α treatment, the 2.4 and 1.6 mm constructs loaded with Ccl2-IL1Ra iPSCs produce similar levels of IL-1Ra, significantly exceeding the IL-1Ra production levels in the 1.6 mm construct loaded with Ccl2-Luc cells shown by dashed lines. (**C**) Fluorescent live/dead labeling (green = live; red = dead) show uniformly distributed live cells throughout the thickness of the 2.4 and 1.6 mm constructs, as well as relatively uniform proteoglycan staining by Safranin-O, scale bars: 500 µm. One-way ANOVA with Tukey’s post-hoc test, n = 4–5/group/timepoint. Different letters (a,b) indicate groups are significantly different from one another (*p* < 0.05).

To determine the effects of construct thickness on IL-1Ra production, we created cylindrical (3 mm diameter) agarose gels with three different thicknesses (2.4 mm, 1.6 mm, and 0.75 mm) (Figure 1A) that were seeded with Ccl2-IL1Ra iPSCs at 2 × 10^6^, 1.5 × 10^6^, and 0.75 × 10^6^ cells, respectively, to achieve the same cell density per construct (100 × 10^6^ cells/mL).

Constructs underwent chondrogenic differentiation for 3 weeks and were then challenged for up to 72 h with a pathophysiologic level of IL-1α (1 ng/mL). All constructs loaded with Ccl2-IL1Ra iPSCs significantly exceeded the IL-1Ra production levels in the 1.6 mm construct loaded with Ccl2-Luc cells (Figure 1B). Release quantity per surface area is also illustrated (Figure S1). Compared to the previously designed 3D woven scaffolds, the 2.4 mm and 1.6 mm constructs released greater levels of IL-1Ra into the media at 24 h although the levels were similar to that of scaffolds at 72 h [6]. Of note, the 2.4 mm construct did not result in greater levels of IL-1Ra released into the media compared to the 1.6 mm construct at 24 or 72 h post IL-1α challenge despite the greater total cell number, suggesting that the overall thickness may reduce IL-1Ra production or release. For both thicknesses (2.4 mm and 1.6 mm), live/dead staining revealed nearly 100% live cells and consistent proteoglycan staining by histology, indicating extracellular matrix (ECM) deposition by encapsulated cells, representative of chondrogenic differentiation and cartilaginous tissue formation (Figure 1C).

### 2.2. Translational Delivery Strategy Using 3D-Printed Molds and Wide Bore Needle Delivery

**Figure 2:**
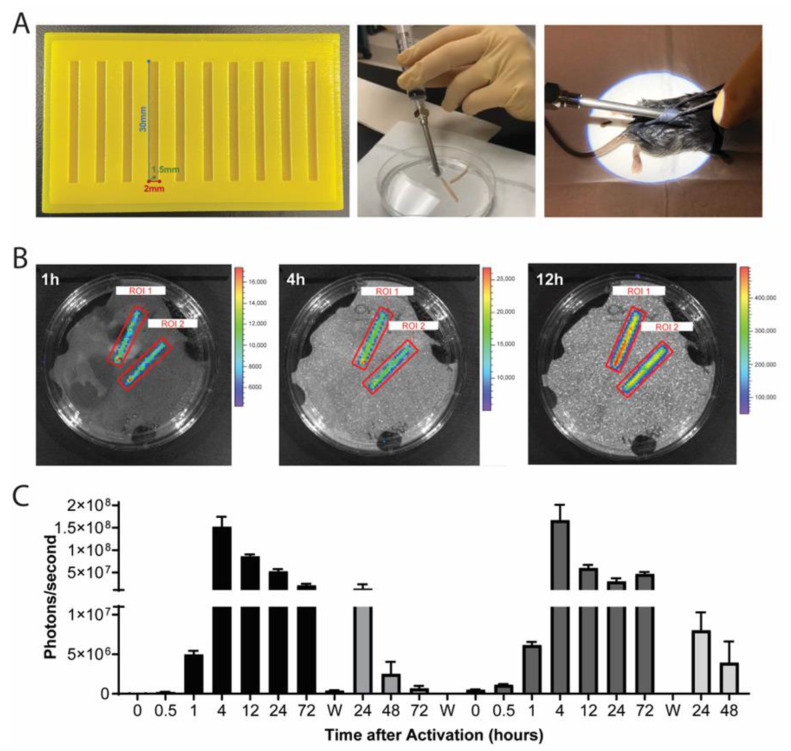
(**A**) Custom-designed molds (left) were 3D-printed to generate cell-laden agarose rod constructs, which were aspirated into a wide-bore needle (middle) for minimally-invasive implantation subcutaneously through a small dorsal incision (right). (**B**) Ccl2-luc agarose rod constructs were challenged with 1 ng/mL IL-1α imaged via IVIS to observe activation over time, shown here is a time course of the same two constructs at 1 h, 4 h, and 12 h post IL-1α challenge. (**C**) Constructs showed robust activation with repeated washout (designated “W”) and reactivation with 1 ng/mL IL-1α. n = 2–6 samples/group/timepoint.

Given these initial results, rectangular agarose rods of mid-size diameter width (2 × 1.5 × 30 mm) were designed to all for injection of an agarose construct using a wide-bore needle. We used a custom 3D-printed mold to generate agarose rods for the minimally invasive blunt needle delivery method to be used in subsequent in vivo assessment (Figure 2A). Rod-shaped constructs were loaded with either 9 × 10^6^ cells of Ccl2-luc or Ccl2-IL1Ra iPSCs to achieve the same cell density as above, and the same therapeutic cell number as used previously [6]. Constructs underwent chondrogenic differentiation [10] for three weeks prior to implantation.

The Ccl2-Luc agarose rod constructs were first evaluated in vitro and shown to respond to IL-1α challenge (1 ng/mL) with strong luminescence (Figure 2B) that was reproduceable after a 72 h washout period (Figure 2C). Early and sustained luminescence was repeatably exhibited within one hour post-challenge. Given these promising activation kinetics, we next evaluated the Ccl2-Luc agarose rods’ response to K/BxN serum challenge [6,26].

### 2.3. Agarose Rod Implants Demonstrate Long-Term Viability and Drug Delivery

Next, the same Ccl2-luc agarose rod constructs were implanted in naïve wildtype mice (5–8 weeks old) and repeated K/BxN serum challenges were administered at 1, 10, and 28 weeks after implantation.

**Figure 3:**
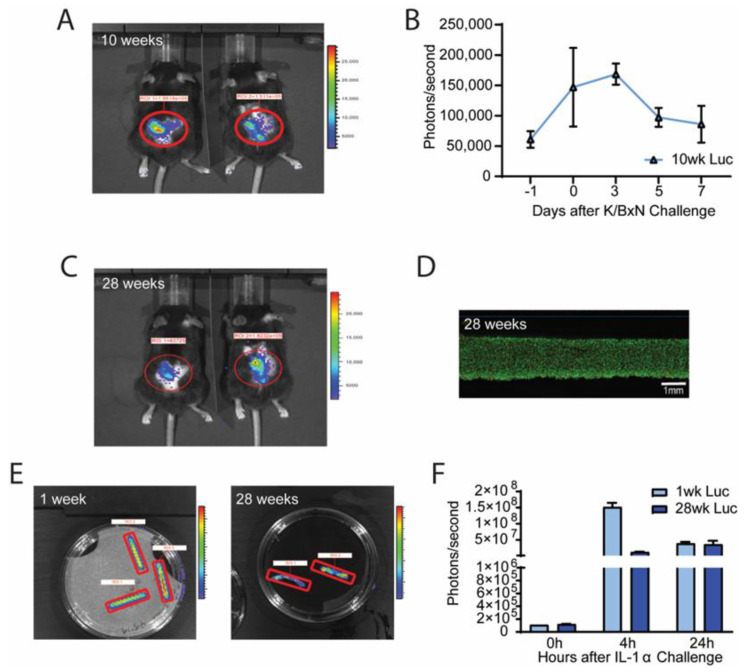
(**A**) Luminescence activity by IVIS 10 weeks after Ccl2-Luc agarose construct implantation indicates that cell activation peaked over the first 3 days after K/BxN serum challenge (**B**). (**C**) Luminescence activity by IVIS from Ccl2-Luc agarose constructs 28 weeks after implantation indicates cell activation after repeated K/BxN challenge. (**D**) Ccl2-Luc constructs demonstrated predominantly live cells upon explantation at 28 weeks, scale = 1 mm. (**E**,**F**) Ccl2-Luc constructs explanted after 1 and 28 weeks of implantation and challenged with 1 ng/mL IL-1α exhibited robust luminescence intensities ex vivo. n = 5–10 animals/group compared by 2-way repeated measures ANOVA and Dunnet’s or Tukey’s post-hoc test, n = 2–8/group/timepoint.

After 10 weeks of implantation, Ccl2-Luc agarose constructs showed strong luciferase activity following a K/BxN serum challenge, demonstrating their long-term viability and responsiveness (Figure 3A,B). Moreover, following 28 weeks of in vivo implantation and a repeated K/BxN serum transfer, Ccl2-Luc constructs continued to show similar level of activity (Figure 3C). This finding was corroborated by a majority of live cells in the Ccl2-Luc constructs explanted after 28 weeks (Figure 3D). Additionally, constructs explanted after 1 week or 28 weeks continued to show robust luminescent intensity when challenged with IL-1α, although we observed a reduced luminescent peak at 4 h in the 28-week explants (Figure 3E,F).

### 2.4. Injectable Anti-Cytokine Therapy Mitigates Arthritis Outcomes in Mice Challenged with K/BxN STA

**Figure 4:**
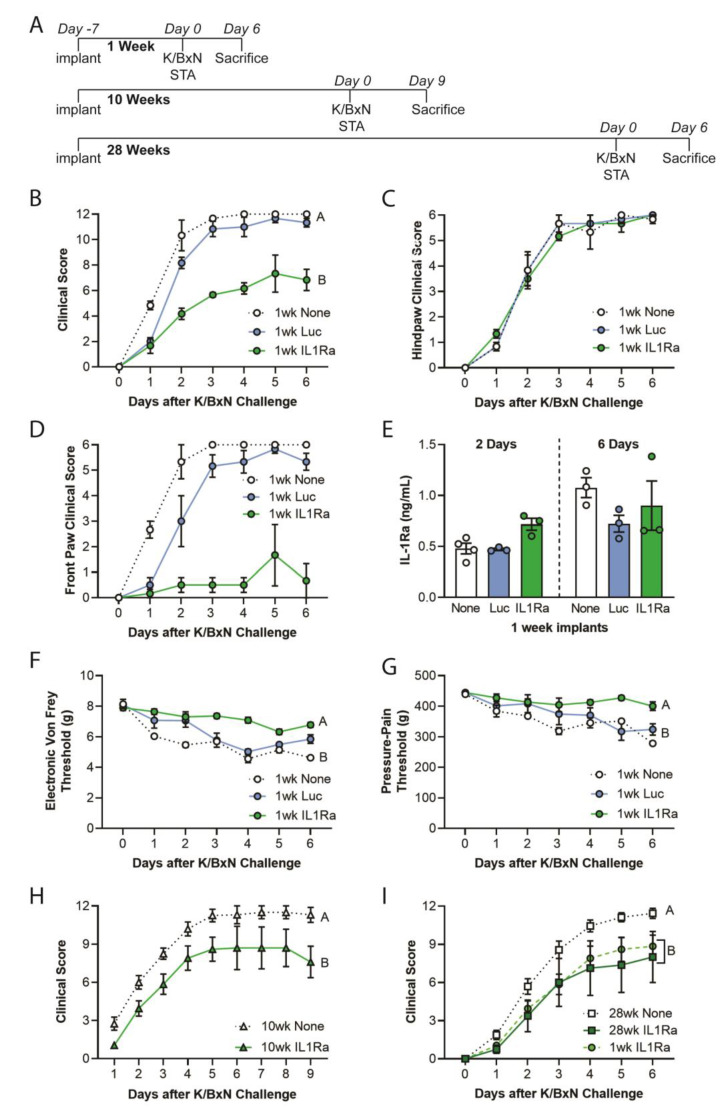
(**A**) Timeline showing in vivo K/BxN serum transfer arthritis (STA) studies at 1 week, 10 weeks, or 28 weeks post-subcutaneous implantation of Ccl2-IL1Ra agarose rods. Clinical scores of K/BxN STA animals 1 week after implantation of Ccl2-luc or Ccl2-IL1Ra agarose rods over 6 days of observation for (**B**) overall clinical scores, (**C**) hindpaws and (**D**) front paws. (**E**) IL-1Ra levels in serum at day 2 post K/BxN serum challenge, as measured by ELISA. IL1Ra constructs significantly mitigated mechanical allodynia by (**F**) Electronic von Frey and (**G**) pressure-pain hyperalgesia. STA overall clinical scores (**H**) 10 weeks and (**I**) 28 weeks after implantation of Ccl2-IL1Ra agarose rods. Note that the degree of disease mitigation observed after 28 weeks is similar to mitigation 1 week after implantation. Different letters indicate significant main effects of group, *p* < 0.05. N = 3–6/group, data were analyzed by 2-way repeated measures ANOVA with Dunnet’s or Tukey’s post-hoc test. Different letters indicate main effect of group *p* < 0.05.

After establishing long-term repeated activity and viability of these agarose rod constructs in vitro, we investigated their therapeutic potential in arthritis mitigation by implanting freshly created Ccl2-IL1Ra or Ccl2-Luc agarose constructs into 5–8 week old mice for various periods, as depicted in the timeline (Figure 4A). Ccl2-IL1Ra constructs significantly mitigated arthritis when mice were challenged with K/BxN serum transfer one week after implantation (Figure 4B). Of note, we did not observe disease mitigation in the hind paws of animals implanted with Ccl2-IL1Ra agarose constructs (Figure 4C) while the front paws were relatively free of disease in these same animals (Figure 4D). Congruent with the clinical scores, we did not observe bony erosions or bone differences on microCT data (Figure S2) or a significant difference in proteoglycan content (via toluidine blue staining) or inflammatory cell infiltration (via H&E staining) of hind paws (Figure S3).

Implanted Ccl2-IL1Ra agarose constructs produced significantly increased serum levels of IL-1Ra at day 2 post K/BxN serum challenge (Figure 4E), which was accompanied by significant mitigation of mechanical allodynia from day 1 (Figure 4F) and pressure-pain hyperalgesia from day 2 (Figure 4G). Cytokine bead array (CBA) showed a marked reduction in IL-1β and a trend in lower levels of TNF-α, IL-6, and MCP-1 in the front paws of animals implanted with Ccl2-IL1Ra rods (Figure S4). Given the strong dependency on IL-1β in this model, these results corroborate the clinical scores (Figure 4 C,D).

In animals implanted with Ccl2-IL1Ra agarose constructs for 10 or 28 weeks and challenged with K/BxN serum, we observed significant reduction in disease severity during the entire period of observation (Figure 4H,I). Moreover, in mice implanted with Ccl2-IL1Ra constructs for 28-weeks, the arthritis clinical scores were similar to those animals challenged with K/BxN serum after one week of implantation, underscoring the long-term activity of these constructs in vivo (Figure 4I).

## 3. Discussion

Rod-shaped hydrogel constructs containing Ccl2-IL1Ra cells subcutaneously implanted in mice using a wide bore needle significantly mitigated K/BxN serum transfer arthritis, with nearly complete abolishment of disease severity in the front paws. A major advance of this study is the demonstration that the induced pluripotent stem cells encapsulated in the agarose hydrogel maintained long-term activity [16], sufficient for disease mitigation even after 28 weeks of implantation. Advantages of this delivery strategy are multifold, including minimally invasive implantation without the need for invasive surgery, while allowing for delivery of higher cell densities in a small, flexible construct. Leveraging the design capabilities of 3D printed resin molds, patient-specific geometries or differential wide-bore needle thicknesses could be generated and used, increasing the translational potential for this approach.

This delivery strategy and design were informed based on initial measurements of IL-1Ra release from cylindrical, disk-shaped implants. In an effort to maximize anti-inflammatory drug delivery, we initially envisioned that larger implants (~2.4 mm thick) encapsulating a higher total number of cells might produce higher levels of anti-inflammatory biologic drug; in actuality, the larger size appeared to hinder IL-1α bioavailability to the cells, thus potentially limiting IL-1Ra synthesis and delivery by the cells. While we established the specific implant dimensions for these studies herein, we anticipate that geometry, design principles, and system intrinsic properties must be assessed in a case-specific manner depending on the use of different anti-inflammatory mediators, cell types, and cell densities. Furthermore, while we based our self-regulating system on the presence of inflammation (i.e., IL-1) as a driver, other inputs may be used to drive transgene expression [27].

We also demonstrated long-term efficacy and reactivation potential of these constructs, which rivals existing implants approved for other applications [28,29,30]. IL-1Ra levels were higher on day 2 in mice that received the Ccl2-IL1Ra constructs compared to untreated animals but this transient spike was sufficient for significant mitigation of disease, suggesting that rapid, early increases in IL-1Ra delivery may be critical for therapeutic efficacy [6]. In the current dataset, it remains unclear why the clinical score in the front paws, but not the rear paws, was completely mitigated in Ccl2-IL1Ra rod-treated animals. The significant disease mitigation was accompanied by marked suppression of IL-1β in the front paws, suggesting differential transport of IL-1Ra from the constructs to these joints specifically. Further studies are needed to address this issue more directly.

### Limitations and Future Work

In the present data, it is unclear whether the endogenous matrix that forms due to chondrogenesis is impairing the release of IL-1Ra from the cell-laden constructs [22], or if other factors may be impairing the treatment efficacy of this approach. We will refine this hydrogel approach to better understand the differences in therapeutic efficacy between the present data and the same cells seeded on a 3D woven scaffold [6].

In previous studies, we observed mitigation of bony erosions with Ccl2-IL1Ra cell-based therapy on scaffolds, which was not observed in the present studies. In fact, there were no major changes detected in the microCT outcomes across all groups. However, there is known variability in bony erosions in K/BxN STA between front and hind paws and from experiment to experiment due to the short duration of the studies. Similarly, we did not observe significant mitigation of proteoglycan loss and immune cell infiltration in hind paw histology, consistent with the clinical scores. Future work aims to tune this hydrogel approach to achieve the same therapeutic effect across all joints, especially given the promising longevity and cell viability (up to 28 weeks post-implantation) that we observed with this system.

## 4. Conclusions

With the advent of cell-based therapies for arthritis and other indications, minimally invasive drug delivery strategies may be adapted and evaluated to accelerate translation to the clinic. We demonstrated that a 3D printed hydrogel construct containing cell-based anti-cytokine therapy delivered via a wide bore needle retained similar levels of therapeutic efficacy and exhibited improved longevity when compared to 3D woven disc-shaped scaffolds that were surgically implanted. This approach allows for custom bioprinting of molds to fit shapes of interest or accommodate other implantation methods for a variety of applications. Cell-based anti-cytokine therapy is a powerful technology that has the capacity to overcome current limitations of biologic drugs, such as dosing and long term drug delivery, when delivered via agarose hydrogels. Future work aims to further refine this approach with alternative hydrogels, a minimally invasive explantation procedure to further improve translational potential.

## 5. Experimental Section/Methods

### 5.1. Cell Culture and Agarose Hydrogels

Using CRISPR-Cas9 genome engineering, mouse induced pluripotent stem cells (iPSCs) were edited to drive expression of Il1rn or luciferase (Luc) under the endogenous macrophage chemoattractant-1 (MCP-1, or Ccl2) locus [1,4] iPSCs were pre-differentiated using micromass culture, and the resulting cells were cultured using expansion media [10] expanded to passage 3. Ccl2-based genome-engineered iPSCs (Ccl2-IL1Ra or Ccl2-Luc) were mixed with 2% agarose hydrogel in molds of three different thicknesses (2.4 mm, 1.6 mm, and 0.75 mm) and punched into 3 mm discs using a biopsy punch. Each disc construct had a total of approximately 2 × 10^6^, 1.5 × 10^6^, and 0.75 × 10^6^ cells, respectively. Constructs were cultured for 21 days in serum-free chondrogenic medium containing Dulbecco’s Modified Eagle Medium–High Glucose (DMEM-HG), non-essential amino acids, 2-mercaptoethanol, ITS+ premix (BD), penicillin-streptomycin (Gibco), 50 μg/mL L-ascorbic acid 2-phosphate, 40 μg/mL L-proline, 10 ng/mL transforming growth factor beta 3 (TGF-β3, R&D Systems) and 100 nM dexamethasone [10,31]. 

We generated custom 3D-printed resin molds to seed the agarose and cell (Ccl2-IL1Ra or Ccl2-Luc) mix into a 2 × 1.5 × 30 mm rod for 90 mm^3^ total volume containing approximately 9 × 10^6^ cells, which could fit inside a blunt 12-gauge needle (diameter 2.15 mm), under sterile conditions. We designed custom 3D-printed polypropylene construct molds using the opensource 3D modeling program Blender (Blender; blender.org). Construct molds were 3D printed using a Fused Filament Fabrication (FFF) Flashforge Creator Pro 3D Printer (Flashforge; Jinhua City, Zhejiang Province, China) and polypropylene filament (Gizmo Dorks; Temple City, CA USA). These rod-shaped constructs were cultured as described above.

The main component of the hydrogel is PBS and agarose. Filtered cells were mixed at a ratio of 1:1 with 4% warmed type VII agarose (Sigma-Aldrich) once it reached 37 °C. The iPSC-agarose mixture was carefully pipetted into a custom 3D printed molds using a p1000 pipette and the mixture allowed to set at room temperature in the mold. The final formulation of agarose is 2%. A custom sterilized wire was used to remove the hydrogel from the mold, and into chondrogenic culture media. These rod-shaped constructs were cultured as described above.

### 5.2. Quantitative Assessment of IL-1Ra

Protein levels of secreted murine IL-1Ra were measured in media via ELISA (Duoset, R&D Systems) at 1:20 dilution in duplicate. Serum levels for IL-1Ra were measured using the Quantikine IL-1Ra ELISA (R&D) at 1:20 dilution in triplicate.

### 5.3. Cell Viability and Proteoglycan Staining

Cell viability in constructs was assessed with the Live/Dead^®^ Cell Viability/Cytotoxicity Kit for mammalian cells (Invitrogen/Molecular Probes, Carlsbad, CA, USA). Constructs were labeled with calcein AM for live cells and ethidium homodimer-1 for dead cells, then imaged using confocal microscopy (LSM 880, Zeiss, Thornwood, NY, USA) [7].

### 5.4. Bioluminescence Imaging 

Constructs were assessed in vitro for bioluminescence using IVIS^®^ imaging in response to cytokine challenge (1 ng/mL IL-1α) over a time period of 48 h, according to previously described methods [6].

### 5.5. K/BxN Model of Inflammatory Arthritis

All procedures were approved by IACUC at Washington University in St. Louis. Ccl2-Luc constructs were injected subcutaneously into C57BL/6 male and female mice. The rods were briefly washed in 37 °C phosphate buffered saline then aspirated using a 10 mL lure lock syringe with a sterilized 12-gauge stainless steel blunt needle. A small subcutaneous incision was made on the dorsal aspect of the mouse, the construct was delivered with the 12-gauge blunt needle, and the small incision was sutured closed with one suture and Vetbond skin glue (3M, St. Paul, MN, USA).

Serum transfer arthritis (STA) flare was induced at 1 week, 10 weeks (2.5 months), and 28-weeks (6 months) post-implantation with 175 µL of K/BxN serum delivered by retroorbital injection [32,33,34]. Ccl2-IL1Ra rod constructs were implanted subcutaneously using the blunt needle approach on the dorsal aspect of C57BL/6 mice (n = 3/treatment group). Control animals received either Ccl2-Luc constructs or no treatment. Disease activity (Clinical Score and Ankle Thickness) were assessed daily for 1 week.

As previously described [6], disease activity (clinical score and ankle thickness) and pain sensitivity (algometry and Electronic Von Frey) were assessed daily. Mice were then sacrificed, and serum and paws were collected for analysis. The severity of disease, termed clinical score, is assigned between 0–3 (0 = no swelling or erythema, 1 = slight swelling or erythema, 2 = moderate erythema and swelling in multiple digits or entire paw, 3 = pronounced erythema and swelling of entire paw; maximum total score of 12) [35]. Digital dial calipers were used to determine ankle thickness daily, which was subtracted from the thickness measured at day zero, on both hind paws [6]. These two values were averaged each day and reported as ankle thickness.

### 5.6. Pain and Behavioral Testing

Animals were acclimatized to the behavioral testing room prior to assessment. Pressure-pain hyperalgesia was assessed daily using a pressure-based analgesiometer (SMALGO, Bioseb, Vitrolles, France) by applying a steady increasing force over the ankle joint until a reaction (shudder or vocalization) from the animal was observed, up to a threshold of 450 g. Three trials of the maximum force were then recorded, averaged, and analyzed.

Electronic Von Frey paw assays were used to determine mechanical allodynia daily. Mice were placed in acrylic cages (12 × 10 × 17 cm high) with a custom-made wire grid floor 15–30 min before testing in a quiet room. During this adaptation period, the hind paws were stimulated 2–3 times, according to previously reported methods [6]. In these experiments, a pressure-meter which consisted of a hand-held force transducer fitted with a 0.5 mm^2^ polypropylene tip (Electronic von Frey anesthesiometer, IITC Inc., Life Science Instruments, Woodland Hills, CA, USA) was used. A tip was applied against the central hind paw and the intensity of the stimulus was recorded with paw withdrawal. The stimulation of the paw was repeated until the animal presented three similar measurements.

### 5.7. Histology

Formalin-fixed paraffin embedded slides of hind paws were prepared at 5 μm thickness, after overnight fixation in 4% paraformaldehyde and decalcification with 10% formic acid (CalEx II). Serial slides were then stained with toluidine blue and hematoxylin & eosin according to previous methods [6].

### 5.8. Cytokine Bead Array Assay

Levels of paw inflammatory mediators were evaluated using a Cytometry Bead Array (CBA, BD Biosciences). Paws were harvested at sacrifice and homogenized in 1 mL of PBS. The paw lysates were cleared by centrifugation and subjected to CBA analysis (Mouse Inflammation Kit and Mouse IL-1β Flex Set) as recommended by the manufacturers.

### 5.9. Statistical Analysis

Sample size was determined based on outcomes of our previously reported studies [6]. Based on an alpha of 0.05 and 80% statistical power (1-β), three animals/treatment group was needed for in vivo studies. Outcomes were evaluated by 2-way ANOVA with post-hoc testing. All assessments and analyses were performed in a blinded manner.

## Data Availability

All data needed to evaluate the conclusions in the paper are present in the paper and/or the Supplementary Materials.

## Supplementary Materials

The following supporting information can be downloaded at: https://www.mdpi.com/article/10.3390/gels9020169/s1, Figure S1: Constructs were loaded with Ccl2-IL1Ra iPSCS at the same densities and stimulated with chondrogenesis media for 21 days. After 24 h and 72 h of 1 ng/mL IL-1α treatment, the 2.4 and 1.6 mm constructs loaded with Ccl2-IL1Ra iPSCs produce similar levels of IL-1Ra, significantly exceeding the IL-1Ra production levels in the 1.6mm construct loaded with Ccl2-Luc cells shown by dashed lines. IL-1Ra secretion was normalized to the construct thickness (2.4mm [surface area = 36.8 mm^2^], 1.6mm [29.2 mm^2^], and 0.75 mm [21.2 mm^2^]). One-way ANOVA with Tukey’s post hoc test, n = 4–5/group/timepoint. Different letters indicate groups are significantly different from one another (*p* < 0.05).; Figure S2. No bone differences between treated and untreated samples were noted by microCT reconstruction, as seen in representative 3D renderings of forepaws (a) and hindpaws (b). No differences were seen in bone mineral density (BMD; g/cm^3^) (c), bone fraction (bone volume/total volume; BV/TV) (d), or bone surface to bone volume (BS/BV) (e). N = 3/group.; Figure S3. No visual differences were noticed between hind paws treated with constructs loaded with Ccl2-Luc cells (Luc, left) and Ccl2-IL1Ra cells (IL-1Ra, right) as seen with representative images of Toluidine blue staining (a) and H&E staining (b).; Figure S4. Cytokine bead array analysis was conducted for the analytes IL-1, IL-6, MCP-1 and TNF-α to detect protein cytokine levels in the paws, n = 3/group, Data were analyzed by 2-way repeated measures ANOVA with Sidak’s post hoc test. Exact p-values are shown above each comparison of the two treatment groups within each limb, n = 3/group.
