# Supplementary material for: Hydrogel Encapsulation of Genome-Engineered Stem Cells for Long-Term Self-Regulating Anti-Cytokine Therapy
Source: Gels. 2023 Feb 20;9(2):169. doi: 10.3390/gels9020169 (PMC9956980; doi:10.3390/gels9020169)
Supplement: Supplementary file 1 [file gels-09-00169-s001.zip › gels-2219378-supplementary.pdf]

Supporting Information

Hydrogel Encapsulation Of Genome-Engineered Stem Cells As An Injectable Self-Regulating Anti-Cytokine Therapy

*Authors: Kelsey H. Collins, Lara Pferdehirt, Leila S. Saleh, Alireza Savadipour, Luke E. Springer, Kristin L. Lenz, Dominic M. Thompson, Jr., Sara J. Oswald, Christine T.N. Pham, Farshid Guilak **

**Corresponding Author*

Supplemental methods: Bone Assessments by MicroCT

To measure bone morphological changes, intact paws were scanned by microcomputed tomography (microCT, SkyScan 1176, Bruker) with a 9 µm isotropic voxel resolution at 50 kV, 500 µA, 980 ms integration time, 3 frame averaging, and 0.5 mm aluminum filter to reduce the effects of beam hardening. Images were reconstructed using NRecon software (Bruker) with 20% beam hardening correction and 15 ring artifact correction. Hydroxyapatite calibration phantoms were used to calibrate bone density values (g/cm^3). The following parameters were calculated using CTAn software (Bruker): bone mineral density (BMD; g/cm^3), bone fraction (bone volume/total volume; BV/TV), and bone surface to bone volume (BS/BV).

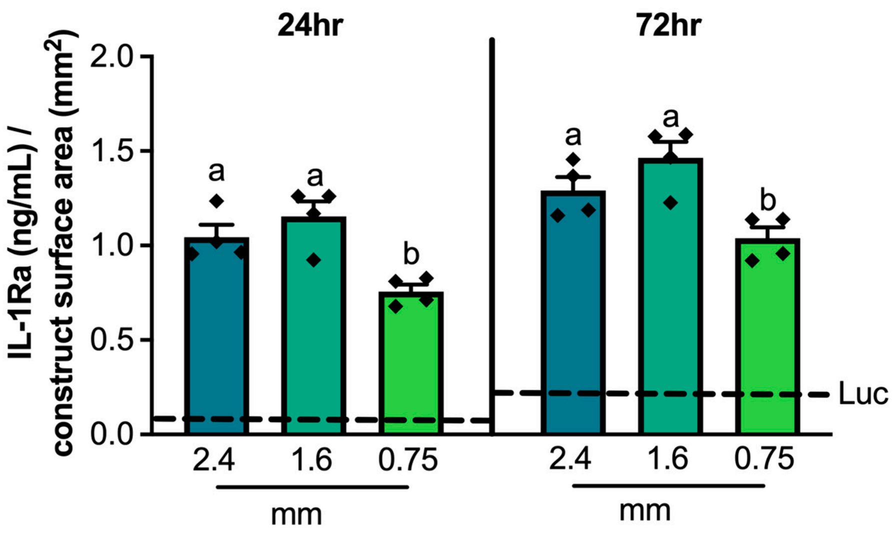

Figure S1. Constructs were loaded with Ccl2-IL1Ra iPSCS at the same densities and stimulated with chondrogenesis media for 21 days. After 24h and 72h of 1 ng/mL IL-1α treatment, the 2.4 and 1.6mm constructs loaded with Ccl2-IL1Ra iPSCs produce similar levels of IL-1Ra, significantly exceeding the IL-1Ra production levels in the 1.6mm construct loaded with Ccl2-Luc cells shown by dashed lines. IL-1Ra secretion was normalized to the construct thickness (2.4mm [surface area =36.8mm^2], 1.6mm [29.2mm^2], and 0.75mm [21.2mm^2]). One-way ANOVA with Tukey's *post hoc* test, n=4-5/group/timepoint. Different letters indicate groups are significantly different from one another ($p<0.05$).

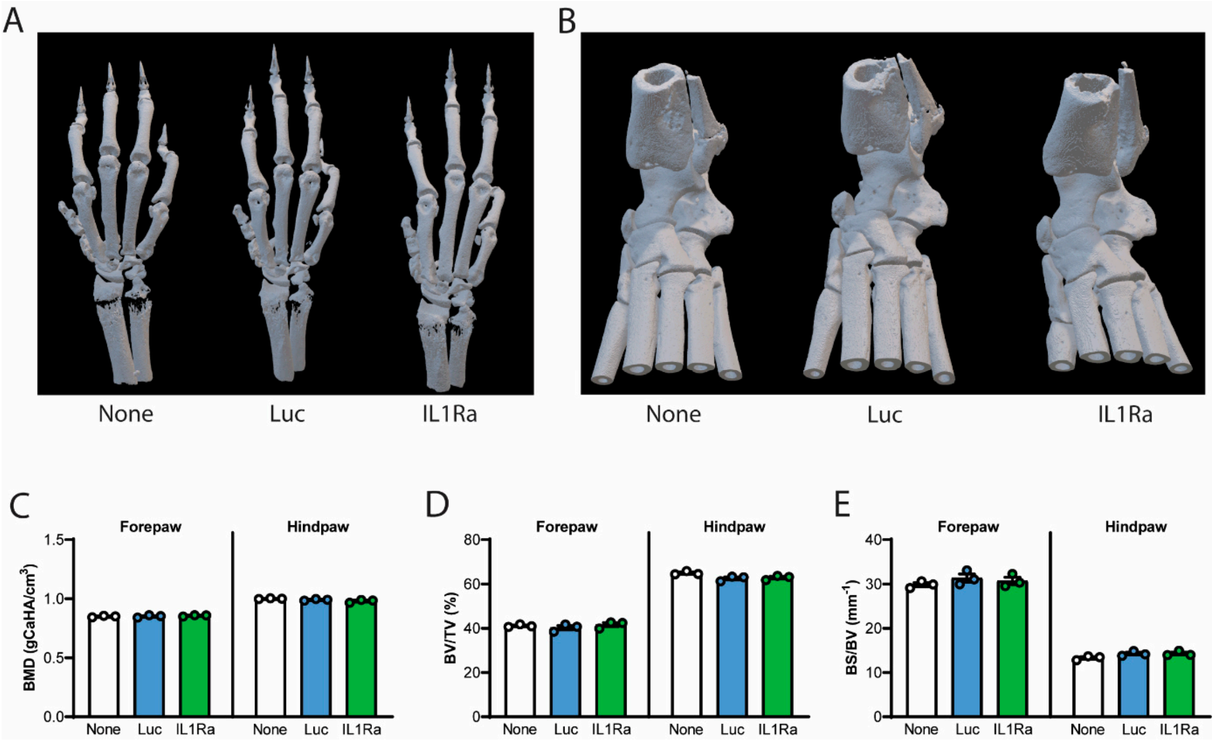

Figure S2. No bone differences between treated and untreated samples were noted by microCT reconstruction, as seen in representative 3D renderings of forepaws (a) and hindpaws (b). No differences were seen in bone mineral density (BMD; g/cm^3) (c), bone fraction (bone volume/total volume; BV/TV) (d), or bone surface to bone volume (BS/BV) (e). N=3/group.

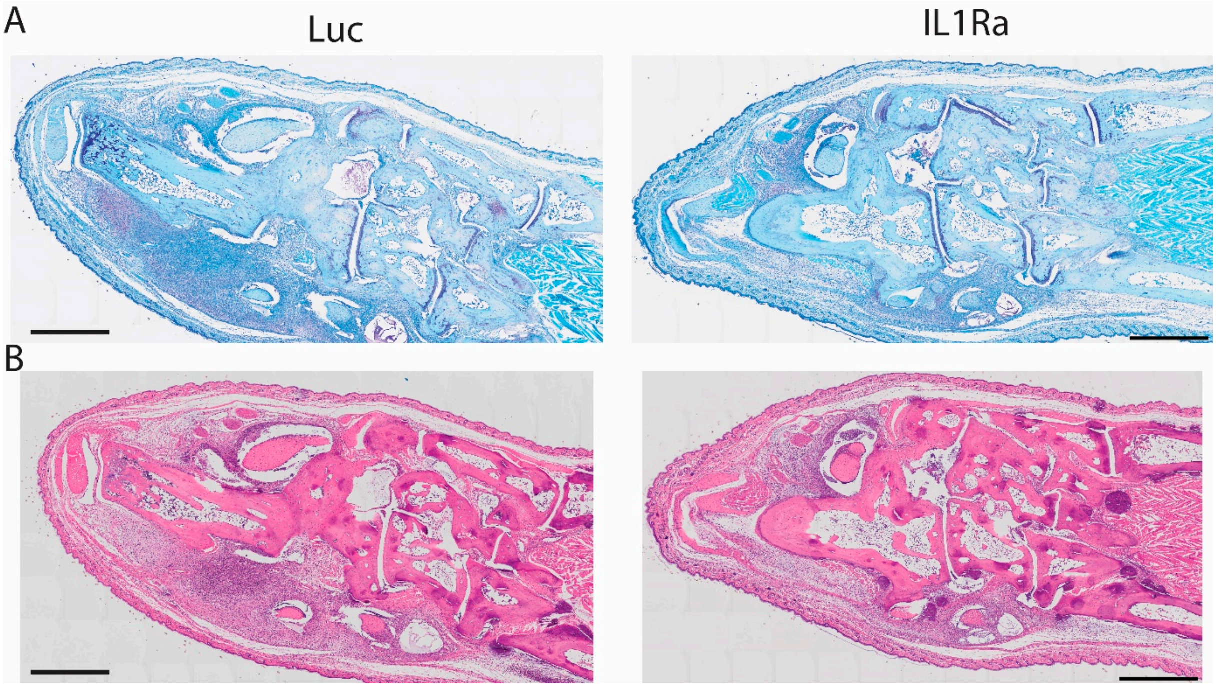

Figure S3. No visual differences were noticed between hind paws treated with constructs loaded with Ccl2-Luc cells (Luc, left) and Ccl2-IL1Ra cells (IL-1Ra, right) as seen with representative images of Toluidine blue staining (a) and H&E staining (b).

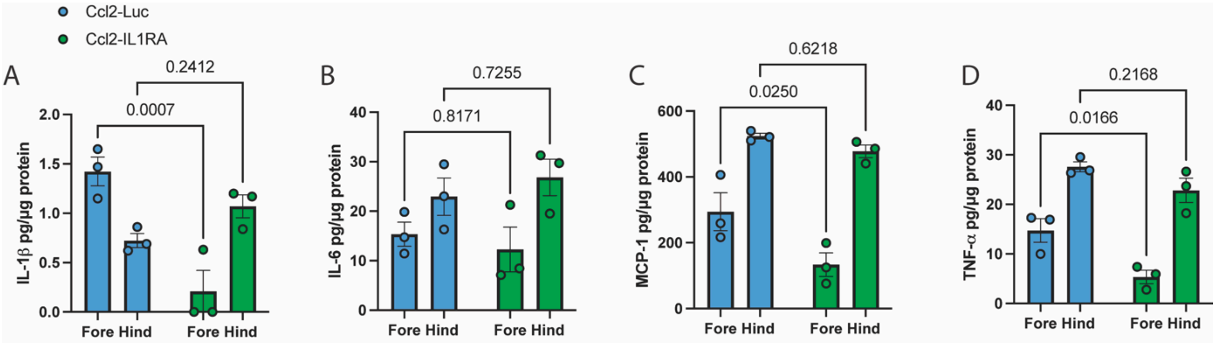

Figure S4. Cytokine bead array analysis was conducted for the analytes IL-1β, IL-6, MCP-1 and TNF-α to detect protein cytokine levels in the paws, n=3/group, Data were analyzed by 2-way repeated measures ANOVA with Sidak's *post hoc* test. Exact p-values are shown above each comparison of the two treatment groups within each limb, n=3/group.
